# Retelling social inequalities in the era of market competition: Review and discussion for sustainable welfare development

**DOI:** 10.3389/fsoc.2023.1085278

**Published:** 2023-02-07

**Authors:** Watchara Pechdin, Chawapon Sarnkhaowkhom, Sudarat Kanthanetr, Martin Pieter Willemse

**Affiliations:** ^1^Thammasat University Research Unit in Social Equity, Faculty of Social Administration, Thammasat University, Bangkok, Thailand; ^2^Department of Health Education and Behavioral Sciences, Faculty of Public Health, Mahidol University, Bangkok, Thailand; ^3^College of Population Studies, Chulalongkorn University, Bangkok, Thailand; ^4^Faculty of Information and Communication Technology, Mahidol University, Nakhon Pathom, Thailand

**Keywords:** social inequality, intergenerational inequality, gender-based inequality, health inequality, education inequality, welfare, sustainable development, market competition

## Abstract

As the prevalence of social inequalities has become increasingly evident, the implementation of social welfare policies in countries across the globe has faced considerable obstacles and has not yielded the desired results. In spite of the fact that social welfare policies are formulated to reduce inequalities in society, the recent increase in inequalities has raised questions about whether or not welfare implementation is appropriate to the social context where resource distributions are dominated by economic structure. Inspired by this, the aim of this paper is to echo contemporary perspectives on social inequality and challenges that have contributed to its development under the economic system of market competition. The contemporary matters arising from social inequalities, which include intergenerational inequality, gender-based inequality, health inequality, and education inequality, are examined in accordance with the context of market competition. This would hopefully enable academicians to timely recognize and address ideological and paradoxical social inequalities and welfare development within their society.

## 1. Introduction

Social inequality has become a fundamental issue of concern for countries across the globe when attempting to secure the well-being of their society members. It has been shown to have a detrimental effect on individuals, families, and communities, leading to lower life satisfaction, increased stress, and poorer physical and mental wellbeing (Bui and Pal, 2022; Klinsrisuk and Pechdin, 2022; Kumar et al., 2022; Pal et al., 2022). Furthermore, these disparities in privilege have led to unequal access to healthcare, education, and economic resources, all of which can negatively impact the quality of life (Jodhka et al., 2017).

Although many countries have initiated various social welfare policies as a key response measure to mitigate the impacts of social inequalities, the effectiveness of those policies still depends on the economic structure of a country, especially those economic structures which promotes competition in the market (Esping-Andersen, 1990; Korpi and Palme, 1998). This is because the market producers tend to minimize their production costs in order to boost their market competitiveness. Therefore, social welfare policies adding costs to the production such as working benefits, pensions, or other concepts related to the workers' well-being might be overlooked. As a result, some of the society members whose hardships are not mitigated for might be left behind in poverty.

This paper sets out to conduct a narrative review aimed at reviving the understanding of four key social inequalities which could contribute to a discussion on welfare development in the context of an economic structure with market competition. The focus of this research has been narrowed down to current issues pertaining to intergenerational inequality, gender-based inequality, health inequality, and educational inequality. These inequalities were selected based on their significant impacts on other critical socio-economic areas such as intergenerational inequality and the addressing of poverty transmission among generations, educational inequality and the addressing of wage and ability fairness, gender-based inequality and the addressing of impacts of gender composition on employment, and health inequality and the addressing of wellbeing and healthcare issues among society.

This review is also expected to enhance the understanding of the development of social welfare policies, particularly when it is dominated by the economic structure of market competition. Although research studies of social inequality and welfare policy exist, the key findings still depend on referred research design, especially regarding their theoretical considerations and lack a more diverse range of perspectives. Therefore, a theoretical framework related to the social actions provided as help under the circumstance of market competition will be presented in this paper. The welfare claims making process will be the central focus of this framework. This discussion on welfare and social inequality will provide a way to analyze how different groups within a society interact with each other when applying for welfare and how some groups are more likely to experience poverty or social exclusion. This could help present a clear construction of welfare development and interactions occurring among various key institutions under the circumstance of market competition.

The main body of this paper is divided into 5 sections. To begin, the theorical considerations associated with welfare development will be offered. Subsequently, the research methodology section is presented. The next section included the results, which selected social inequalities are reviewed. This is followed by the discussion section which discusses the relationship between economic structure and inequality together with an investigation on the paradoxes of implementing welfare policies. Some concluding remarks and some key takeaways are included in the last section.

## 2. Theoretical consideration

Numerous difficulties, possibly related to the social context, the involved actors, and the economic structure of a country, have plagued theories of welfare development. This study highlights an approach offered by Drover and Kerans (1993), whose development is based on the concept of social action and focuses specifically on claimsmaking. The intention of including this approach was to contribute to a discussion on the sustainability of social welfare development by examining the current conditions of welfare development, in particular the claiming process which is considered the first step of welfare development.

Claimsmaking or Claims-making refers the process through which groups of people (such as advocacy or social movement organizations, community groups, law makers, or reporters) convince others (such as government officials, or the general public) that special support should be given to a certain group of people. Regarding the claimsmaking process, institutional order is considerable. Although the only potential responses to claims are explicit acceptance or rejection, in the majority of cases claims are neither accepted as presented nor rejected outright (Drover and Kerans, 1993). The responses are often influenced by the power of dominant groups. As long as the hegemonic order is recognized, it is rare for dominant group's claims to be questioned or reframed as counterarguments completely. In contrast, claims that are not completely and satisfactorily met by the hegemonic order will be marginalized. From this it can be inferred that institutional orders could influence the response to the claimsproposal in society.

Looking into the claimsmaking process, Drover and Kerans (1993) explained that the claimsmaking process should comprise of three steps. Firstly, claimants have to make their claims publicly for others helping them and arranging themselves into a group in order to reveal and empower their needs. It might be accepted or rejected by the hegemonic groups, but acknowledgment of their needs from the hegemony is what is expected. The next step is for the individuals to harmonize themselves with the group identity of the group based on which they are making a claim and polish their own identity centered on it. They tend to strategize or organize their mind toward that community. For example, they attempt to share the same psychological mind, thoughts, and social norms. This is to strengthen their identity with that certain group. In the last step, concerted acts constitute a social movement toward advocating their claims.

The claimsmaking process is an essential initial step for individuals or groups to express their needs in order to protect their interests and advance their well-being. By strategically engaging the public in an effort to draw attention to their cause, those affected by inequality are able to take proactive steps to address their grievances and create meaningful change. Through this process, members of society can leverage the power of collective action to advocate for their rights, increase representation, and ultimately reduce inequality they experience.

## 3. Method

### 3.1. Scope of work

The fundamental goal of this research study is to review social inequalities within the economic system of market competition, where a market producer will produce output through cost minimization. Consequently, for this research, a narrative review was constructed that focused on four thematic areas of social inequalities, namely, intergeneration inequalities, educational inequalities, gender-based inequalities, and health inequalities. Although the narrative review only highlights four thematic areas of social inequalities, researchers looking at other inequality issues might be willing to adopt parts of this theorical discussion for further consideration.

### 3.2. Data collection procedure

Data was collected from several major online databases including Scopus, Web of Sciences, and Google Scholar. The period the collection of research focused on was 2012–2022. The selected references had to have followed the original peer-review process. The procedure of collecting the data comprised of the following 3 steps:

- *Step 1 Identifying area of interest:* This step is to find documents which are in line with the main objective of the study. The documents were looked for by searching for general keywords. The contents of the abstracts were considered during the selection process.
- *Step 2 Narrowing down the document to cope with the thematic areas:* This step focused on narrowing down the content of articles for review in accordance with the thematic areas of the study, which in this study are the four major inequalities. The content was prescreened based on the thematic keywords.
- *Step 3 Extending of relevant references/material:* In this step the selected references from Step 2 were reviewed and other selected references/materials were examined to see if they supported the thematic area in question which support the selected references. Some references did not fall into the 2012–2022 time period. This process was followed to ensure the correct interpretation and comprehensive communication of information.

All three steps of the data collection procedure are summarized in Table 1.

**Table 1:** Data collection procedure.

**Step 1** **Providing a broad scope through major thematic keywords**	**Step 2** **Narrowing down the content through thematic keywords**		**Step 3** **Checking references extensively**
	**Social inequalities**	**Thematic keywords**	
Welfare, social inequalities, sustainable development, national policies, market competition, free market	Intergenerational Inequality	Intergenerational inequality, wealth inequality, intergenerational transmission, life-discourse	Searching for other references extensively in accordance with their contributions to the selected references from Step2
	Educational Inequality	Educational inequality, education attainment, child welfare	
	Gender-Based Inequality	Gender employment, gender gap, family policies, parenthood penalties	
	Health Inequality	health inequalities, health at work, health barriers, health policies, health promotion	

Summarized by authors.

Moreover, the issue of the biased selection of references was taken into consideration as a blind selection among the research team was conducted. In step 2, each author was asked to individually read and choose the ones of interest from the found references. Following this, the selected references by the authors were compared. When similar chosen references were found, they were checked extensively in Step 3. On the other hand, unmatched references were brought up for individual discussion and deeper examination prior to being forwarded to Step 3. This was believed to have eliminated the author's bias when selecting the references.

### 3.3. Selected studies for review of literatures

**Figure 1:**
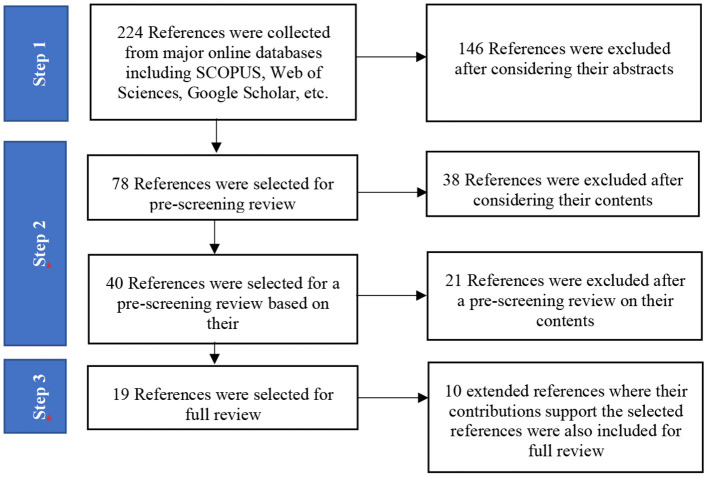
Process of selecting references for review.

From searching for a broad scope of keywords, 224 original references were collected after deleting some of the duplicate pieces of work. In the final stage when the contributions to the thematic area of the research project were considered, 19 references were chosen for full review. The process of selecting references was summarized and presented in Figure 1.

Details of the selected references and their extensions are summarized in Table 2.

**Table 2:** Summary of selected references.

**Social Inequalities**	**Selected References**	**Extended References/materials**
Intergenerational inequality	Corak, 2013; Torche, 2014; Saez and Zucman, 2016; Christophers, 2018; Durlauf and Seshadri, 2018; Agupusi, 2019	Erikson and Goldthorpe, 2002 Organization for Economic Co-Operation and Development (OECD), 2006
Educational inequality	Fusarelli, 2015; Lathapipat, 2018; Herbaut and Geven, 2020; Tadesse and Muluye, 2020	Lanza, 2010, Coleman, 2019, Organisation for Economic Co-operation Development, 2019
Gender-based inequality	Signorelli et al., 2012; Andreotti et al., 2013; Georgiadis and Christopoulos, 2017; Brandts, 2021, Paweenawat and Liao, 2022	Bettio, 2008, Organisation for Economic Co-operation and Development (OECD), 2020 World Economic Forum, 2020
Health inequality	Mackenbach, 2012; Russell et al., 2013; Fujishiro et al., 2021; Das, 2022	Carrillo et al., 2011 Organisation for Economic Co-operation and Development (OECD), 2019

Summarized by authors.

## 4. Results

This section provides an overview of the emergence of four key social inequalities in society. It also highlights the emergent issues of social inequality mostly found among the working class of an economy, which will pave the way to discuss its relationship to economic structure in Section Discussion on social welfare development: economic structure, paradox, and sustainability.

### 4.1. Intergenerational inequality

Since it is likely that inequalities have been carried down from one generation to the next, the number of intergenerational inequality studies has increased as it has become a more essential research area. Intergenerational inequality, which might be defined as the inheritance of inequality (Erikson and Goldthorpe, 2002), could be the outcome of low levels of intergenerational mobility. The Organisation for Economic Co-operation and Development (OECD) [Organization for Economic Co-Operation and Development (OECD), 2006] defined intergeneration mobilities as “the extent to which some key characteristics and outcomes of individuals differ from those of their parents”. This can be simplified as “the extent to which differences in parental generation are passed on to the next generation”. Therefore, it was hypothesized that intergenerational inequality would decrease when the family background had a significant impact on the adult outcomes of young people, and vice versa when the family background had a less significant impact.

The impact of market competition and intergenerational inequality has been widely studied. The literature suggests that market competition can lead to an increase in this type of inequality, as those with more resources are able to access better basic needs. This can in turn lead to an intergenerational cycle of inequality, as children of wealthier parents are more likely to succeed. This is not only unfair, but can also lead to social and economic stagnation, as those at the bottom are unable to move up (Durlauf and Seshadri, 2018). More importantly, a recent study found that when there is more competition in the labor market, it rapidly leads to greater inequality between generations (Saez and Zucman, 2016). This was due to the fact that competition leads to higher wages for the most skilled workers whose family's endowment enables them, while simultaneously leading to lower wages for the less skilled. This, in turn, leads to greater inequality in earnings and wealth between generations.

Other studies have also found that intergenerational inequality has been on the rise in developed countries over the past few decades as a result of increased market competition. The rich have gotten richer while the poor have gotten poorer. This trend has led to an increased inequality in opportunities and decreased intergenerational mobility. Corak (2013) argues that income inequality is a major contributor to these trends. He argues that income inequality decreases intergenerational mobility because it limits the ability of people to move up the income ladder. Moreover, income inequality creates conditions for individuals in disadvantaged positions, pressuring them to choose and act in ways that perpetuate their classes' status quo (Erikson and Goldthorpe, 2002). Especially, this inequality constructs strong barriers to disadvantaged families having opportunities in attaining a quality education (Torche, 2014), which ultimately discourages them to pursue high skilled training. This promotes a poverty circle since it motivates them to remain status quo in society (Agupusi, 2019).

However, there is a suggestion to focus the discussion on intergenerational transmission when looking at the contemporary findings (Christophers, 2018). This is owing to the fact that the fundamental relevance of generational relations to the dynamics of intergenerational inequality tends to relate to economic transfers rather than intergenerational differences. As suggested by Christophers (2018), the ability of members of a family to transfer their wealth from older to younger generations tends to cause patterns of intra-generational inequality; therefore, it is preferable to investigate how generations are interconnected as compared to measuring the nominal distance they are apart. More particularly, his findings suggested that differences between generations is less important than the transfer of wealth between other generation when identifying trends in generational inequality. This dynamic process is unlikely to be effectively understood unless the transmission process is emphasized.

In summary, the majority of scholars believe that intergenerational inequality is a structural process from one generation to the next, with particular emphasis on wealth accumulation. Instead of focusing on the factors that set them apart from other classes, current research suggests that policies aimed at closing this gap should emphasize the reducing of the transmission process.

### 4.2. Educational inequality

Education has been recognized as one of the human investments in human capital. However, inequalities, which come in the form of problems and causes, constrain and limit people to gain accessibility to that. Long debates have pointed out that the educational inequality could be a result of the family background and personal characteristics, geographical accessibility, a household's wealth status, or financial obligations (Lathapipat, 2018). It might also extend to race and socioeconomic classes across some parts of the globe (Fusarelli, 2015).

In recent years, the relationship between economic structure and educational inequality has come under increased scrutiny. A number of studies have shown that an economic structure with market competition can lead to increased educational inequality, with some groups being left behind and others benefiting disproportionately. A growing body of evidence supports that economic structure, especially when it comes to capitalism, is a major driver of this phenomenon. Altogether with the Organization for Economic Co-operation Development (Organisation for Economic Co-operation Development, 2019), the study found that, in countries with higher levels of income inequality, students from low-income families are more likely to be concentrated in lower-quality schools and to have lower test scores. This is significantly contributed to by the economic structure of the country. Additionally, some studies point out that one of the key mechanisms through which an economic structure can increase educational inequality is through the way it structures opportunities for advancement (Lanza, 2010). In a market competition system, those who are able to take advantage of opportunities and climb the ladder of success are often the ones who come from more privileged backgrounds. This can create a situation where the children of the wealthy are more likely to access a higher quality of education than the children of the poor (Coleman, 2019).

Furthermore, educational inequalities were more clearly revealed during the COVID-19 pandemic. According to Tadesse and Muluye (2020), educational inequalities existed massively in developing countries as a result of the tremendously unequal access to educational resources and technology. This was especially the case with regard to remote learning during the closure of the schools during the pandemic as there was a lack of parental awareness, academic devices, and curriculum materials for students to effectively study in this environment. Other than that, the pandemic also attributed to disparities at higher educational level, as such education was less likely to be participated in by a student with a low socioeconomic background (Organisation for Economic Co-operation Development, 2019). Herbaut and Geven (2020) summarized that the key barriers during the pandemic faced by disadvantaged students in higher education included unmet financial needs, unsuitable academic preparation, lack of information, and behavioral deficits. All these were caused by inequalities in resource accessibilities due to economic inequality resulting from the economic structure.

There are a number of reasons why an economic structure with market competition, in particular capitalism, potentially increases educational inequality. As addressed, market competition is associated with a higher level of income inequality, which can lead to increased segregation by income and social class. When the economic structure comes to be capitalism, it is seen that capitalist societies tend to have more unequal access to resources, including education. This is because in capitalist societies, resources are allocated according to market principles, which means that those with more money are able to buy better quality resources. Together, all referred references demonstrate the complex ways in which the competition in the market contributes to educational inequality. While there is no easy solution to this problem, a better understanding of the root causes of this inequality is a necessary for the completion of the first step of social development.

### 4.3. Gender-based inequality

As researched by Organization for Economic Co-operation and Development (OECD) [Organisation for Economic Co-operation and Development (OECD), 2020], the difference between men's and women's median earnings in 2018 varied from 3.4% in Luxembourg to 34.1% in South Korea. This massive gap demonstrated global concerns over gender-based inequalities for opportunities to seek a valuable income. Indeed, it is undeniable that these disparities are largely caused by the underlying market structure which are often characterized by discriminatory occupational practices, perpetuated by entrenched social norms and the prejudiced attitudes of employers (Andreotti et al., 2013).

The literature on market competition and gender-based inequality is abundant and growing. Despite this, there is still a lack of consensus on how competition affects gender inequality. Some scholars argue that competition between firms leads to a “race to the bottom” in terms of wages and working conditions, which disproportionately affects women (Bettio, 2008; Georgiadis and Christopoulos, 2017). The debate over the impact of market competition on gender inequality is unlikely to be resolved anytime soon, but the existing literature provides some useful insights into the mechanisms through which competition may affect gender relations (Brandts, 2021). In particular, competition may affect gender inequality through its impact on the firms' hiring and promotion practices, as well as its impact on wages and working conditions. This is likely due to a number of reasons, such as women being paid comparable less for the same type of work, and women mainly working in sectors that are more vulnerable to competition. For example, women are more likely to work in the service sector, which is often one of the first sectors to be affected by a recession (Signorelli et al., 2012; Brandts, 2021). When economies contract, people are less likely to spend money on non-essential services. This means that service sector workers, in particular female laborers, are more likely to lose their jobs or see their hours cut back.

Recently, some research on women's earning stated that the significant gap in earnings was a result of the difference in the number of household jobs and care responsibilities that women had compared to men. As evidenced by the World Economic Forum (World Economic Forum, 2020), this disproportionate burden is an underlying factor that contributes to the financial disparity between women and men. There is no country in which men dedicate the same amount of time to unpaid labor, such as domestic and volunteer work, as women. In contrast, in many countries, women continue to devote multiple times as much time as men to these activities. Even in countries where he number was lower, such as Norway and the United States, women spent almost twice as much time as men on housework, which they did not receive a salary for. The consequence is that this deprives their opportunities to learn, increase their skills, or find competitive employment. In addition, when taking parenthood penalty into consideration, several pieces of research reveal that there is a significant gap in ages between females who are parents and those who are not. This is supported by Paweenawat and Liao (2022), whose key findings pointed out that the average annual salary of a woman without children is higher than that of a woman with children, regardless of whether or not the woman is married. This shows that having children has a detrimental effect on a woman's earning potential, adding another layer of inequality to gender differences.

By focusing on the correlation between gender and income opportunities, in a nutshell, it is possible for gender-based issues to generate an additional layer of inequality when a circumstance or set of situations discourages opportunities for those who are concerned by it. This is especially the case when it comes to issues that are associated with the parenthood penalty, which can result in an increased burden on childcare and household responsibilities for women, which in turn leads to the widening of the wage gap between men and women. As a result, several governments have begun implementing a variety of social welfare policies in an effort to close this gap. One such illustration of this is the encouragement and facilitating of duties between men and women.

### 4.4. Health inequality

In recent years, there has been a growing body of evidence that market competition can lead to increased health inequality as well. A number of studies have shown that when markets are more competitive, there is a greater gap between the health outcomes of the rich and the poor. Health inequality could be defined as “differences, variations, and disparities in the health achievements of individuals and groups”, according to Kawachi et al. (2002). However, thematic discussions on inequalities in health fundamentally include disparities in access to primary care doctors, hospitalization, and preventative services such as cancer screening, influenza vaccination, or dental treatment. This inequality might be caused by demographical conditions, such as where people are born, grow up, live, work and get older. It is acknowledged that a good health influences the chances of someone in the labor market, educational achievements, and community engagements, and increases the chances of people finding employment, being more productive, and improving one's livelihood, while rising health disparities would diminish these chances. To significantly reduce health inequalities, more focus should be put on the individual, psychological, and cultural determinants of health as suggested by academic contributions (Mackenbach, 2012).

Inequalities in health are increasing in all regions around the world, even in developing nations and welfare states. The Organisation for Economic Co-operation and Development (OECD), 2019 reported evidence from EU countries that indicated that unmet care requirements were mostly concentrated among lower income groups, particularly in the lowest income quintile, and that poor households had more difficulty affording care when they accessed the system. According to their statistics, 26% of those with the lowest incomes did not receive necessary treatment owing to the high cost, compared to 8% of those with the greatest incomes not receiving the necessary treatment Moreover, it can be said that unmet financial demands are prevalent among lower income groups in all nations, not only in the developing countries. This shows that the lower income groups are more likely to incur expensive health care costs.

It has been observed that recent research has put the mainstream focus of health inequalities on healthcare accessibility, particularly related to barriers to accessibility. Carrillo et al. (2011) introduced the Health Care Access Barriers Model (HCAB), which offers a taxonomy and a practical framework for the classification, analysis, and reporting of barriers to healthcare that were connected to differences in health care. This model was developed as a result of the authors' efforts to address the problem of health care disparities. Under this model's framework, it was evidenced that barriers to healthcare accessibility consisted of three main components: *financial*—cost of care and health insurance status barriers; *structural*—including the transportation to the health care facility, continuity of care, waiting time or operating hours at the health care facility; and *cognitive*— awareness of prevention facts, health literacy, understanding of treatment, and communication barriers. Although this important HCAB conceptualization has been referenced in various studies worldwide for its development framework addressing social inequality, there are different views and suggestion on its context. For example, in recognition of Russell, Humphreys (Russell et al., 2013), the study highlighting issues in health barriers in rural and remote areas suggested that healthcare should first be available to people and in the correct geographical t before other aspects of access could be considered. It could ensure common accessibility whether there are differences in the population's characteristics and needs or not.

Due to the consideration of these cognitive barriers, the number of research contributions aimed at generating knowledge to reduce health inequalities keeps rising. According to Fujishiro, Ahonen (Fujishiro et al., 2021) who studied work and health equity through the lens of political economy, occupational health research would organizational change to promote more health and health equity for its workforce, by offering specific health information for increasing awareness at work. Therefore, researchers play an important role in institutional change toward health equity. There is support on this matter. Under a structure of market competition, Das (2022) echoed that it is necessary not only to understand the social determinants of health better, but also produce the knowledge among common people to try and get a better health. This would decrease health inequality in the workplace.

## 5. Discussion on social welfare development: Economic structure, paradox, and sustainability

### 5.1. Economic structure and social welfare policy

The structure of the economic system strongly correlates with social inequalities. This section discusses the social inequalities when the economic system of a country shifts to market competition, which has become a powerful economic structure this century. As mentioned in previous sections, academicians have increasingly focused on the role played by market competition in the exacerbating of social inequalities. Competition among firms for market share and profits has led to higher inequality as firms seek to maximize their own returns rather than invest in the long-term well-being of their employees or society. This can result in lower wages, fewer benefits, and fewer opportunities for advancement, particularly for those at the bottom of the income ladder.

The power of market producers i.e., private ownership has caused challenges with regards to the claims making process. As noted, increasing the welfare for laborers or wage earners will increase the production costs of the private enterprises. Therefore, employers are mostly discouraged to offer additional benefits to their employees. As a result of this, it could be inferred from the contributions of the selected references that most of the welfare benefits for laborers tend to be introduced by the government rather the enterprises. This is due to the fact that the government has difficulties forcing the private sectors to issue welfare policies as it would impact the employment structure in the labor markets. However, the success in claims making for welfare by the working class could be achieved if there is significant power to support their advocacy. As evidenced by Scandinavian countries, the working class could be successful with their claim-making with the backing of a national labor party whose members are largely from the middle-to-lower classes in the labor market (Esping-Andersen, 1990).

In addition, it could be also observed that promoting a welfare state for the working-class encounters challenges in a number of different areas in developing countries, especially when wanting to enforce the private employer to provide welfare for their employees. Pechprasert (2021) argued that the struggle in introducing welfare for private employees in developing countries was due to lack of representatives of the working class in institutional decision-making. The reason for this is that in many developing countries, most of the political representatives in national government are backed by the private or upper class of society, and as a result, they compromise with private enterprises, particularly when establishing pension regimes or providing additional welfare through national employment laws and regulations.

Obviously, it could be concluded that unfavored contributions of market competition resulting in a lack of support toward wage earners leads to extend inequalities between the classes in society, in particular between the working class and middle-to-upper class such as their employers. This lack of financial and benefit support may lower intergenerational mobility which in turn could explain intergenerational inequality. This coincides with the findings of Erikson and Goldthorpe (2002) that inadequate conditions for those in disadvantaged positions potentially drive them to remain status quo within the class. This is observed when wage earners in a working class receive fewer financial gains and welfare benefits in an industry where they spend most of their time receiving an income. Children subsequently inherit less capital to receive a future return or other benefits. This is because most of their parental income will be spent on covering their everyday expenses. This also attributes to fewer educational opportunities offered by the parents to their children, paving the way to educational inequality as pointed out by Torche (2014) and Lathapipat (2018). As a result, they will likely find it difficult to move across a societal class.

This problematic circumstance can also explain the difficulties in promoting welfare to reduce the parenthood penalty in developing countries where the welfare state has not taken off yet. Few true working-class representatives in the national government yield the power to persuade employers to offer benefits to parents in need, so only standard benefits as defined by labor law are offered. Consequently, a lack of progress is seen when promoting benefits, such as parental leave to reduce gender inequality in a workplace in developing countries, as mentioned by Paweenawat and Liao (2022). With regards to health inequality, it can be clearly seen that it is also caused by limited financial resources, according to the HCAB model (Carrillo et al., 2011). In non-welfare states as well as developing countries driven by a market competition structure, an employer mostly compromises health assurance for their employees with the national healthcare system. Heath welfare policies, as example by Dorsey and Topol (2016) or Das (2022), are not in their favor as it could create additional costs. As a result, those working-class workers can access only basic health assurance provided by the state/nation government, but most health services are facing several challenges and there are barriers to health access.

In conclusion, it is foreseen based on previous studies that the market competition system can lead to challenges, especially with regards to providing equal welfare policies. In non-welfare states including developing countries, welfare policies are normally implemented by national governments rather than capitalist classes or companies in the private sectors. The cost of production is the main reason. Looking into historical welfare development, scholars have proven that the success in welfare development is influenced by the strength of the institutional setting. Representatives from the working class in the government could be key players to advocate for working-class welfare claims, and determine whether or not welfare policies are successfully being implemented.

### 5.2. Paradox in resource redistribution

Nonetheless, it should be emphasized that whether or not based on social justice fundamentalism or moral norms, there is a paradox between the introduction of social welfare policies and inequality under the economic structure of market competition. Inequalities in society may be exacerbated rather than diminished by social welfare policies, particularly when these policies are influenced by social structure, redistribution preferences, rules and regulations, and a further ineffective recruitment process of disadvantaged people. Then, there is also the fact that national social welfare policies are executed by the country's political system, so some social privileges may be then only granted to a certain society, who have a similar identity to the national identity. This can be observed in some countries. For instance, the Bumiputera privilege in Malaysia (Gomez, 2012), or citizen laws in Myanmar against some ethnicities can be put forward as evidence.

In addition, Korpi and Palme (1998) indicated that the institutions of the welfare state play a crucial role in generating the redistribution paradox. This is as a result of conflicts of interest among different groups in society because each group has its own interests and coalitions, which in turn has consequences regarding the size of budgets available for the distribution of benefits and the level of equality achievement. Therefore, this limitation might lead conflicts during the redistributive process of benefits.

Additionally, another concern is caused by distribution preferences, also known as populism policies. This is because they lead to social marginalization, while undermining social equality. A populist policy is one of the factors that leads to social exclusion among people who have different opinions or are different from the dominant group (Babajanian and Hagen-Zanker, 2012). This stipulates the inequality concept of “deservingness”, favoring dominant groups over those in need. A country with more inequality is likely vulnerable to populism (Pástor and Veronesi, 2021). In particular, when people are on the margin of a certain group, their claims might be overlooked. It can be observed globally that populism is widening inequality in various aspects, such as former president Trump's policies toward Hispanics, Brexit and household income earnings, acceptance of same sex marriage in some countries.

Both paradoxes showcase that a social welfare policy has the power to create inequalities in society. When looking at the perspective of well-being development, these paradoxes must be recognized when executing these welfare policies.

### 5.3. Social welfare and sustainability

The level of social exploitation in the advanced capitalist world, which poses a threat to sustainable development, may come as a surprise to those who are not acquainted with the research on social welfare and market competition. Since the post-war period, growth in productivity and GDP has been associated with the achievement of economies of scale, with the goal of increasing production and consumption (Büchs and Koch, 2017). Products and services are produced while reducing costs as much as possible in an effort to increase market competitiveness. As a result, it is quite unlikely that employees will be provided with attractive welfare regimes in addition to the country's existing labor laws by their employers. Consequently, it is anticipated that this situation may lead to various forms of social inequality.

Moreover, when considering the welfare and inequality development, it is clear that the sustainability of the welfare society has been challenged by an aging society. As discussed in Section 5.1 and 5.2, the government institution is expected to act as a major driver of welfare developments and bear the brunt of the costs associated with their issuance. Consequently, social inequalities might be widened when fiscal budgets are reduced. In reaction to these budget reductions, tax collections are seen as a major source of government income. Especially progressive taxation policies are expected to help equalize income inequalities in the capitalist world (Duncan and Sabirianova Peter, 2016; Stephenson, 2018).

However, it is concurred among global experts that the rising proportion of older persons in society has posed tax revenue issues, hence raising grave worries about the longevity of welfare benefits (Morel and Palme, 2018; Dundar Aravacik, 2019; Gal and Bleikh, 2019; Ko, 2020). The aging population can pose a significant structural challenge to fiscal sustainability in two primary areas including (i) a declining working population, which includes taxpayers; and (ii) rising government spending on aged-related services especially healthcare ones (Yoshino et al., 2019). Therefore, there are recommendations to pay greater attention to the concerns of an aging society and to develop appropriate countermeasures such as extending the tax collecting period by providing older individuals with decent employment (Asavanirandorn et al., 2022). This is in agreement with Yoshino et al. (2019) who stated that efforts should made to create fiscal sustainability while the growing population is aging by putting a central focus on three reformation areas which are the quantity and quality of the labor supply, public finances, and pensions. This is attributable to the fact that a rising population that is becoming older necessitates new forms of working conditions, a new taxation system, and a different system for pensions. Consequently, all of these reforms are necessary for macroeconomics and fiscal sustainability in order to cope with the medium- and long-term consequences.

Furthermore, tax regimes that promote green economy growth and encourage the development of industries for environmental and ecosystem conservation may be implemented. Environmental challenges affect all people, particularly with regards to the increasing health inequality among the poor, and have become increasingly pressing since the Rio Summit in 1992 and the Kyoto Protocol in 1997 (Diniz, 2007). Existing studies have been devoted to documenting, understanding, and inaugurating financial environmental measures, such as pollution taxes, and carbon credit, in response to the negative effects of environmental exploitation caused by inefficient production and consumption in a competitive economy (Magnin, 2018; Suša, 2019; Long et al., 2020). Consequently, these measures might lead to a potential initiative for collecting national revenue for fiscal welfare management in compensation for the reduction in individual income tax received from a growing aging society. Other than that, it also ensures environmental sustainability by establishing optimal consumption of environment resources from the market producers.

Concisely, circumstances of population structure, which impact national budget collection for implementing social welfare regimes, and conditions of sustainable environment resource, which affect the well-being and quality of life of society members, exist for the sustainable development of social welfare. National policymakers have to take these concerns into consideration.

## 6. Conclusion

Observations in this review on social welfare policies and inequalities in society have echoed several positive and negative perspectives caused by the competition structure. Positive perspectives support that social welfare policies would reduce disparities among disadvantaged and underprivileged people, particularly when they have difficulties advocating and promoting their rights and livelihoods. This inclusive social welfare could be obviously seen in welfare states which entailed representatives from the working class. In developing countries, a lack of national resources, financial resources in particular, can lead to paradoxes in redistribution preferences and exploit social inequality. This attributes to negative perspectives that social welfare policies are probably returned as a tool for political means, especially through the issuing of populist policies that benefit only its supporters and its beneficiaries. As a result, it worsens social inequalities.

Based on contributions looking at recent matters of social inequality and debates on social welfare development under the economic structure of competition, it can be concluded that social welfare policies cannot succeed in the decreasing of social inequalities in a concise manner until its paradoxes are properly recognized and documented. This is because the issuing of welfare among the working-class people is essentially influenced by dominant powers of the economic structure, especially when political decisions have to be made in a capitalist society. The reason for this is that the issuing of welfare policies will lead to difficulties due to an increase in production costs. This situation establishes critical challenges to executing social welfare policies, and the equality development in society. The policymakers are advised to bear this matter in mind.

Upon considering the trajectories of countries that have successfully launched welfare states, one mechanism from the government that would support advocacy in claiming welfare in the workplace could be increasing the number of representatives of the working class on the boards of companies/enterprises. Although several countries have issued labor laws pushing companies to establish a labor union in the workplace, they are mostly unsuccessful as the low proportion of working-class representatives has no real influence on the board when negotiating about welfare as evidenced by Pechprasert (2021). With this in mind, it is recommended that policymakers address this issue and seek effective mechanisms to empower their advocacy.

## Author contributions

WP and CS contributed to the conceptualization, design, and methodology of this study, while also being responsible for the searching of relevant studies, the preparing and constructing of the original draft, and the writing and revision of this manuscript. SK and MW contributed to the searching for relevant studies and the writing and revision of this manuscript. All authors contributed to the article and approved the submitted version.

## References

[B1] Agupusi P. (2019). The effect of parents' education appreciation on intergenerational inequality. Int. J. Educ. Dev. 66, 214–222. 10.1016/j.ijedudev.2018.09.003

[B2] Andreotti A. Mingione E. Pratschke J. (2013). Female employment and the economic crisis: Social change in Northern and Southern Italy. Eur. Soc. 15, 617–635. 10.1080/14616696.2013.836406

[B3] Asavanirandorn C. Pechdin W. Trang N. T. Q. (2022). Identifying factors influencing productivity of older workers in service sector: a case study in pilot companies in Thailand. Behav. Sci. 12, 268. 10.3390/bs12080268 36004839 PMC9405377

[B4] Babajanian B. Hagen-Zanker J. (2012). Social protection and social exclusion: an analytical framework to assess the links. Background Note. London: ODI.

[B5] Bettio F. (2008). “Occupational segregation and gender wage disparities in developed economies: Should we still worry?,” in Frontiers in the Economics of Gender (England, UK: Routledge), 183–207.

[B6] Brandts J. (2021). Competition and Gender Inequality: A Comprehensive Analysis of Effects and Mechanisms. Barcelona, Spain: Barcelona School of Economics.

[B7] Büchs M. Koch M. (2017). Postgrowth and Wellbeing: Challenges to Sustainable Welfare. Berlin, Germany: Springer.

[B8] Bui N. Pal I. (2022). “Assessing the impact of COVID-19 to tourism and adaptation strategies: a case study in Thua Thien Hue, Vietnam,” in Pandemic Risk, Response, and Resilience. (Amsterdam, Netherlands: Elsevier), 383–398.

[B9] Carrillo J. E Carrillo V. A Perez H. R Salas-Lopez D Natale-Pereira A Byron A. T. (2011). Defining and targeting health care access barriers. J. Health Care Poor Underserved 22, 562–575. 10.1353/hpu.2011.0037 21551934

[B10] Christophers B. (2018). Intergenerational Inequality? Labour, Capital, and Housing Through the Ages. Antipode 50, 101–121. 10.1111/anti.12339

[B11] Coleman J. S. (2019). “Family involvement in education,” in School, family and community interaction (England, UK: Routledge), 23–37.

[B12] Corak M. (2013). Income inequality, equality of opportunity, and intergenerational mobility. J. Econ. Perspect. 27, 79–102. 10.1257/jep.27.3.79

[B13] Das R. J. Capital, capitalism and health. Crit. Sociol. . (2022). 28:08969205221083503. 10.1177/08969205221083503

[B14] Diniz E. M. (2007). Lessons from the Kyoto Protocol. Ambiente sociedade 10, 27–38. 10.1590/S1414-753X2007000100003

[B15] Dorsey E. R. Topol E. J. (2016). State of telehealth. N. Engl. J. Med. 375, 154–161. 10.1056/NEJMra1601705 27410924

[B16] Drover G. Kerans P. (1993). New Approaches to Welfare Theory. Cheltenham, United Kingdom: Edward Elgar Publishing

[B17] Duncan D. Sabirianova Peter K. (2016). Unequal inequalities: Do progressive taxes reduce income inequality? Int. Tax Public Finance 23, 762–783. 10.1007/s10797-016-9412-5

[B18] Dundar Aravacik E. (2019). Social Policy and the Welfare State. Public Economics and Finance Available from: https://www.intechopen.com/books/public-economics-and-finance/social-policy-and-the-welfare-state 10.5772/intechopen.82372

[B19] Durlauf S. N. Seshadri A. (2018). Understanding the great gatsby curve. NBER Macroecon. Ann. 32, 333–393. 10.1086/696058

[B20] Erikson R. Goldthorpe J. H. (2002). Intergenerational inequality: A sociological perspective. J. Econ. Perspect. 16, 31–44. 10.1257/089533002760278695

[B21] Esping-Andersen G. (1990). The Three Worlds of Welfare Capitalism. https://www.google.com/search?biw=1536&bih=670&q=princeton$+$nj&stick=H4sIAAAAAAAAAOPgE-LUz9U3MMm1KDFR4gAxc7KKq7S0spOt9POL0hPzMqsSSzLz81A4VhmpiSmFpYlFJalFxYtYeQqKMvOSU0vy8xTysnawMu5iZ-JgAADg2bhQWgAAAA&sa=X&ved=2ahUKEwjepcT32Nj8AhVWEFkFHRQnB3MQmxMoAHoECF0QAg Princeton, NJ: Princeton University Press.

[B22] Fujishiro K. Ahonen E. Q. de Porras D. G. Chen I. C. Benavides F. G. (2021). Sociopolitical values and social institutions: Studying work and health equity through the lens of political economy. SSM – Popul. Health 14, 100787. 10.1016/j.ssmph.2021.100787 33898729 PMC8056461

[B23] Fusarelli L. D. (2015). Child Welfare, Education, Inequality, and Social Policy in Comparative Perspective. Peabody J. Educ. 90, 677–690. 10.1080/0161956X.2015.1087779

[B24] Gal J. Bleikh H. (2019). The welfare system: An overview. Taub Center Blog 2019, 23.

[B25] Georgiadis T. Christopoulos G. (2017). Gender inequalities in labour market outcomes: Evidence for Greek regions before and throughout the crisis. Int. J. Manpower. 38, 675–695. 10.1108/IJM-11-2015-0198

[B26] Gomez E. T. (2012). Targeting horizontal inequalities: Ethnicity, equity, and entrepreneurship in Malaysia. Asian Econ. Papers 11, 31–57. 10.1162/ASEP_a_00140

[B27] Herbaut E. Geven K. (2020). What works to reduce inequalities in higher education? A systematic review of the (quasi-) experimental literature on outreach and financial aid. Res. Soc. Stratif. Mob. 65, 100442. 10.1016/j.rssm.2019.100442

[B28] Jodhka S. S. Rehbein B. Souza J. (2017). Inequality in Capitalist Societies. England, UK: Routledge

[B29] Kawachi I. Subramanian S. V. Almeida-Filho N. (2002). A glossary for health inequalities. J. Epidemiol. Commun. Health 56, 647. 10.1136/jech.56.9.647 12177079 PMC1732240

[B30] Klinsrisuk R. Pechdin W. (2022). Evidence from thailand on easing covid-19's international travel restrictions: an impact on economic production, household income, and sustainable tourism development. Sustainability 14, 3423. 10.3390/su14063423

[B31] Ko H. (2020). Measuring fiscal sustainability in the welfare state: fiscal space as fiscal sustainability. Int. Econ. Econ. Policy 17, 531–554. 10.1007/s10368-019-00453-2

[B32] Korpi W. Palme J. (1998). The paradox of redistribution and strategies of equality: welfare state institutions, inequality, and poverty in the western countries. Am. Sociol. Rev. 63, 661. 10.2307/2657333

[B33] Kumar A. Tuladhar N. Pal I. (2022). “Demystifying impacts of cyclone Amphan 2019 amid COVID-19 pandemic in West Bengal, India,” in Civil Engineering for Disaster Risk Reduction (Berlin, Germany: Springer), 461–478.

[B34] Lanza F. (2010). Behind the Gate: Inventing Students in Beijing. New York, NY: Columbia University Press.

[B35] Lathapipat D. (2018). Inequalities in educational attainment,” in Education in Thailand: An old elephant in search of a new mahout, ed. G.W. Fry. Singapore: Springer, 345–372.

[B36] Long M. A. (2020). Food insecurity in advanced capitalist nations: A review. Sustainability 12, 3654. 10.3390/su12093654

[B37] Mackenbach J. (2012). The persistence of health inequalities in modern welfare states: The explanation of a paradox. Soc. Sci. Med. 75, 761–769. 10.1016/j.socscimed.2012.02.031 22475407

[B38] Magnin E. (2018). Varieties of Capitalism and Sustainable Development: Institutional Complementarity Dynamics or Radical Change in the Hierarchy of Institutions? J. Econ. Issues 52, 1143–1158. 10.1080/00213624.2018.153601727025539

[B39] Morel N. Palme J. (2018). Chapter 38: Financing the welfare state and the politics of taxation,” in The Routledge Handbook of the Welfare State, B. Greve, Editor. (England, UK: Routledge), 400–409. 10.4324/9781315207049-41

[B40] Organisation for Economic Co-operation Development (2019). How Does Socio-Economic Status Influence Entry Into Tertiary Education? Paris, France: OECD Publishing.

[B41] Organisation for Economic Co-operation and Development (OECD) (2020). LMF1.5 Gender pay gaps for full-time workers and earnings differentials by educational attainment. Available from: https://www.oecd.org/els/LMF_1_5_Gender_pay_gaps_for_full_time_workers.pdf (accessed on May 22, 2022).

[B42] Organisation for Economic Co-operation and Development (OECD) (2019). Health for Everyone? Social Inequalities in Health and Health Systems. OECD Health Policy Studies. Paris: OECD Publishing.

[B43] Organization for Economic Co-Operation and Development (OECD) (2006). Society at a glance, OECD social indicators. Paris, France: Organization for Economic Co-Operation and Development. https://www.google.com/search?biw=1536&bih=670&q=Paris&stick=H4sIAAAAAAAAAOPgE-LUz9U3MLRILixU4gAxTQtLsrS0spOt9POL0hPzMqsSSzLz81A4VhmpiSmFpYlFJalFxYtYWQMSizKLd7Ay7mJn4mAAAEnNX21TAAAA&sa=X&ved=2ahUKEwjUxvre2dj8AhXPFFkFHfi8AAIQmxMoAHoECGgQAg

[B44] Pal I. Sukwanchai K. Bhuridadtpong A. Pal A. (2022). “Impacts of pandemic on education sector in Thailand,” in Pandemic Risk, Response, and Resilience (Amsterdam, Netherlands: Elsevier), 457–469.

[B45] Pástor L. Veronesi P. (2021). Inequality aversion, populism, and the backlash against globalization. J. Fin. 76, 2857–2906. 10.1111/jofi.13081

[B46] Paweenawat S. W. Liao L. (2022). Parenthood penalty and gender wage gap: Recent evidence from Thailand. J. Asian Econ. 78, 101435. 10.1016/j.asieco.2021.101435

[B47] Pechprasert N. (2021). Building Thailand to the Welfare State: From Pridi Banomyong to the Present, ed. W. Luangmanee. Bangkok: Semsikkhalai.

[B48] Russell D. J. (2013). Helping policy-makers address rural health access problems. Aust. J. Rural Health 21, 61–71. 10.1111/ajr.12023 23586567

[B49] Saez E. Zucman G. (2016). Wealth Inequality in the United States since 1913: Evidence from Capitalized Income Tax Data ^*^. Q. J. Econ. 131, 519–578. 10.1093/qje/qjw004

[B50] Signorelli M. Choudhry M. Marelli E. (2012). The Impact of Financial Crises on Female Labour. Eur. J. Dev. Res. 24, 413–433. 10.1057/ejdr.2012.3

[B51] Stephenson A. V. (2018). The impact of personal income tax structure on income inequality for Belgium, Bulgaria, Germany, Lithuania, and Poland: A comparison of flat and graduated income tax structures. Atlan. Econ. J. 46, 405–417. 10.1007/s11293-018-9601-y

[B52] Suša O. (2019). Global dynamics of socio-environmental crisis: dangers on the way to a sustainable future. Civitas - Revista de Ciências Sociais 19, 315. 10.15448/1984-7289.2019.2.31969

[B53] Tadesse S. Muluye W. (2020). The impact of COVID-19 pandemic on education system in developing countries: a review. Open J. Soc. Sci. 08, 159–170. 10.4236/jss.2020.810011

[B54] Torche F. (2014). Intergenerational mobility and inequality: The Latin American case. Ann. Rev. Sociol. 40, 619–642. 10.1146/annurev-soc-071811-145521

[B55] World Economic Forum (2020). Global Gender Gap Report 2020. Geneva, Switzerland.

[B56] Yoshino N. Kim C. J. Sirivunnabood P. (2019). Aging population and its impacts on fiscal sustainability. Aging Societies.

